# Comparison of knowledge and attitudes toward cancer among African Americans

**DOI:** 10.1186/1750-9378-4-S1-S15

**Published:** 2009-02-10

**Authors:** Natalie Thurman, Camille Ragin, Dwight E Heron, Renae J Alford, Cecile Andraos-Selim, Cornelius Bondzi, Jamila A Butcher, Jamison C Coleman, Charity Glass, Barbara Klewien, Aerie T Minor, Diana J Williams, Emanuela Taioli

**Affiliations:** 1 Department of Epidemiology, University of Pittsburgh Graduate School of Public Health, Pittsburgh, PA, USA; 2 Division of Cancer Prevention and Population Science, University of Pittsburgh Cancer Institute, Pittsburgh, PA, USA; 3 Department of Biological Sciences, Hampton University, Hampton, VA, USA; 4 Department of Radiation Oncology, University of Pittsburgh Cancer Institute, Pittsburgh, PA, USA; 5 Department of Epidemiology and Biostatistics, Downstate School of Public Health, State University of New York, USA

## Abstract

**Background:**

It has been noted that the African American population in the U.S. bears disproportionately higher cancer morbidity and mortality rates than any racial and ethnic group for most major cancers. Many studies also document that decreased longevity is associated with low educational attainment and other markers of low socioeconomic status (SES), both of which are prevalent in African American communities across the nation. Evidence suggests that this phenomenon may be due to attitudes that reflect a lack of knowledge surrounding facts about cancer awareness and prevention. This study was designed to yield data concerning the general population's attitudes toward cancer, taking into consideration racial and/or socioeconomic differences in the population studied.

**Results:**

Two hundred and fifteen subjects participated in the survey, of which 74% (159/215) defined themselves as African-American, 20% were White, and 6% were of other races. While only 38% of the study population was able to identify at least 5 risk factors associated with cancer, a lower proportion of African Americans identified at least 5 risk factors than whites (34% vs. 53%, p = 0.03). In addition, a slightly higher percentage of African Americans (10%) were not aware of the definition of a clinical trial when compared to whites (8%, p > 0.1). Of those aware of the definition of a clinical trial, African Americans were more reluctant to participate in clinical trials, with 53% answering no to participation compared to 15% of whites (p = 0.002).

**Conclusion:**

When comparing results to a similar study conducted in 1981, a slight increase in cancer knowledge in the African American population was observed. Our results suggest that while knowledge of cancer facts has increased over the years amongst the general population, African Americans and lower income populations are still behind. This may affect their risk profile and cancer early detection.

## Background

Marked disparities exist in cancer incidence and mortality among minorities when compared with the majority Caucasian population nationwide [1]. African Americans, in particular, have disproportionately higher incidence and mortality rates than any racial group for most cancers in the U.S. African American men have the highest death rates for lung, colon, and prostate cancer; and black women have the highest death rates for colon and breast cancer [2].

Even with an abundance of materials readily available about cancer, cancer is still poorly understood in African American communities. Coupled with lower levels of education and related lower occupational status and income, African Americans often face barriers that result in lower obtainment of health information. In cases where information is made available, studies have shown that prevention materials are often ineffective [3]. Many cancer prevention strategies are aimed at the majority class i.e. white, educated, middle-class individuals [3]. The same messages that may resonate with this population do not necessarily carry the same influence for African American underserved populations [3,4]. Many cancer education materials are culturally insensitive and irrelevant to many underprivileged African Americans.

Studies have shown that medically underserved ethnic minorities have, in general, less knowledge about cancer than do whites [1]. A 2005 national population-based US study found that over 25% of Hispanics and 18% of African Americans (compared with 14% of whites) believed there was nothing they could do to reduce their risk of cancer [5]. Another study using data from the National Interview Health Survey examined the association between established risk factors for cancer (e.g. age, sex, education, income, family, smoking, alcohol use, body mass index, and physical activity) and perceived cancer risk in a sample of 32,374 asymptomatic adults age 18 and older. Researchers found that racial and ethnic minorities were less likely to perceive cancer risk when compared with non-Hispanic white populations [6]. Less knowledge and poor perception of cancer facts contributes to the higher morbidity and mortality rates observed in minority, especially African American communities.

The purpose of this study was to examine knowledge and perceptions related to cancer prevention and awareness in adults ages 18 and over. In particular, we are interested in determining the racial differences in knowledge and perception of cancer.

## Methods

### Survey design

The study survey consisted of 12 questions aimed at assessing the participant's knowledge of cancer screening and cancer risk. Sociodemographic variables including gender, age, race/ethnicity, education level achieved, and yearly household income were collected. Participant's names and other identifiable information were not recorded in this survey. The study protocol was approved by the University of Pittsburgh Institutional Review Board.

### Survey distribution

Subjects from the adult general population, 18 years and older, were invited to participate in this study. Individuals from the general public in the McKeesport and Pittsburgh, PA areas were asked to participate in the survey at various locations where events sponsored by the University of Pittsburgh Medical Center (UPMC) were being held. This included church events, health fairs, and community festivals. In addition, permission was granted to hand out surveys to UPMC staff and volunteers of the Hillman Cancer Center in Pittsburgh, PA.

Surveys completed by the general public from the Hampton and Richmond, VA area were administered by student volunteers at Hampton University as part of a collaborative training program between the University of Pittsburgh Cancer Institute and Hampton University. The students administered the surveys to the general public at various locations throughout the Hampton, VA and Richmond, VA communities (such as laundry mats, police stations, churches, and supermarkets).

Approximately 400 surveys were distributed across each location and a total of 215 surveys were collected and analyzed.

### Statistical analysis

Comparisons of proportions between African-American and Caucasian subjects were performed after adjusting for income and education level using STATA SE (version 10) statistical software (StataCorp LP, College Station, TX).

A cluster analysis was performed on a total of six questions that were true or false and cancer screening questions. Each question fell into one of four categories, early detection knowledge (one question qualified), risk/screening knowledge (three questions qualified), treatment perception (one question qualified), and cancer perception (one question qualified). Each cluster that was created was then given a score based on level of knowledge and positive perception in each question category. For early detection knowledge and risk/screening knowledge questions, if >50% of subjects chose the correct answer, the cluster received a score of one, correlating with more knowledge. If <50% of subjects chose the correct answer, the cluster received a score of zero, correlating with less knowledge. The same scoring system was then used for treatment perception and cancer perception questions. Each cluster was then characterized based on race/ethnicity, education level, income level, gender, and age. Cluster analyses were performed using SAS version 9.2 statistical software.

## Results

### Study population

Demographic characteristics are presented in Table 1, according to self-reported race. African Americans comprised the majority of the study population (74%), while Whites accounted for 20%. The remaining 6% consisted of other races. When taking into account education level, there is a statistically significant difference between African Americans and Whites who have completed high school or less (13% vs. 0%, p-value = 0.01). A statistically significant higher percentage of Whites (100%) have completed a post-secondary education than African Americans (87%; p < 0.0001). In relation to household income, a greater percentage of African Americans (62%) have a yearly household income of less than $35,000.00 compared to Whites (37%; p = 0.003). The percentage of individuals with a yearly household income greater than $55,000.00 is also statistically different between Whites and African Americans (44% vs. 21%, p = 0.003).

**Table 1:** Demographic variables stratified by race/ethnicity.

	N(%)	African-Americans N (%)	Caucasians N (%)	Other N (%)	p-value*
**Study Population** **Gender**	215 (100)	159 (74)	43 (20)	13 (6)	
Male	77 (35)	57 (36)	13 (30)	7 (54)	
Female	138 (65)	102 (64)	30 (70)	6 (46)	
**Age**					
18–35	105 (49)	79 (50)	19 (44)	7 (54)	
36–55	62 (29)	47 (30)	11 (26)	4 (31)	
56+	48 (22)	33 (21)	13 (30)	2 (15)	
**Education Level**					
High school or less	23 (11)	21 (13)	_	2 (15)	0.01
Post-secondary education	192 (89)	138 (87)	43 (100)	11 (85)	<0.0001
**Annual household income**					
Less than $35, 000	123 (57)	99 (62)	16 (37)	8 (62)	0.003
$35, 000 to $55, 000	34 (16)	26 (16)	8 (19)	_	
Greater than $55, 000	58 (27)	34 (21)	19 (44)	5 (38)	0.003

*p-value shows statistically significant differences between African-Americans and Caucasians; N = number.

### Assessments of risk factor knowledge and cancer perception

Participants were first asked to identify risk factors associated with cancer. Table 2 depicts the results according to race, and adjusted for annual household income and education level. Whites were better able to identify at least five risk factors related to cancer occurrence than African Americans (p-value = 0.03). Analysis of individual answers show that Whites were better able to identify age (65% vs. 33%, p = 0.0005) and unhealthy diet (77% vs. 53%, p = 0.01) as risk factors than African Americans.

**Table 2:** Identification of risk factors stratified by race/ethnicity.

	N (%)	African Americans*	Caucasians*	p-value§
**Study Population**	215 (100)	159 (74)	43 (20)	
**Answer Choices**				
Smoking	182 (85)	150 (94)	42 (98)	
Family History of Smoking	188 (87)	134 (84)	43 (100)	
Heavy Alcohol Consumption	105 (49)	72 (45)	23 (53)	
Age	86 (40)	52 (33)	28 (65)	0.0005
Obesity	103 (48)	73 (46)	23 (53)	
Unhealthy Diet	127 (59)	85 (53)	33 (77)	0.01
				
**Selected at least 5 risk factors**	82 (38)	54 (34)	23 (53)	0.03

*Prevalence adjusted for annual household income and education level. §p-value shown only when there is a statistically significant difference between African Americans vs. Caucasians.

Table 3 summarizes survey responses to selected questions. These proportions were also calculated after adjusting for income and education. The first question inquired about treatment methods for individuals diagnosed with cancer. Whites were better able to identify all three forms of standard treatment than African Americans (60% vs. 17%, p = 0.0039).

**Table 3:** Selected responses to survey stratified by race/ethnicity

	Total pop. (n/N)	African Americans*	Caucasians*	p-value§
**What kind of treatment is available for individuals diagnosed with cancer?**				
*Answer: Identified radiation, chemotherapy, and surgery*	156/215	17% (58.4–73.9)	60% (76.0–97.1)	0.0039
**What feeling comes to mind when you hear the words "clinical trial?"**				
*Answer: Hopeful*	116/215	53% (45.6–61.2)	62% (45.9–75.1)	
*None, have never heard of this*	20/215	10% (6.0–16.3)	8% (2.7–22.9)	
**If asked to participate in a clinical trial testing a new form of treatment, would you participate?**				
*Answer: No*	148/215	53% (43.1–63.9)	15% (5.4–35.0)	0.002
**If you are a male, have you been screened or plan to be screened for prostate cancer?**				
*Answer: Yes*	30/54	60% (45.5–74.0)	50% (11.8–88.0)	
*Answer: No*	24/54	39% (26.0–54.5)	50% (12.0–88.2)	
**Where can you find additional information regarding facts about cancer?**				
*Answer: Identified Doctor or Nurse, Cancer Information Services, American Cancer Society*	165/215	77% (70.1–83.6)	83% (67.5–92.3)	

*Prevalence adjusted for annual household income and education level. §p-value shown only when there is a statistically significant difference between African Americans vs. Caucasians. n = number of subjects that provided a specific answer; N = number of total population

Subjects were next asked to express the feeling that comes to mind when mentioned the words "clinical trial". Over half of the entire study population, 54%, answered hopeful. African Americans were slightly more likely to answer "none, have never heard of this" than Whites (10% vs. 8%, p > 0.1). Participants were then asked if they would participate in a clinical trial. A higher proportion of African Americans subjects answered "no" compared to White subjects (53% vs. 15%, p = 0.002).

Male subjects were asked if they have or would consider being screened for prostate cancer. Adjusting for income and education, 50% of the population of White males answered yes, while 60% of African American men answered yes. Fifty percent of White males answered no and 39% of African American men answered no. Overall, subjects noted mostly young age (59%) and lack of insurance (29%) as reasons for not being screened (data not shown).

Female subjects were asked if they have or would consider being screened for breast cancer. The entire population of White females answered yes, while the majority of African American females (91%) answered yes, 6% answered no, and 3% answered not sure. The majority of the population noted lack of insurance (67%) and fear of results (17%) as reasons for not being screened (data not shown).

Subjects were then asked to identify sources for additional information regarding cancer information. The majority of the population in both Whites (83%) and African Americans (77%) identified all three corrected sources (Table 3).

### Cluster analysis

The cluster analysis was then performed. A total of five clusters were created; Table 4 displays each cluster scores based on the model previously outlined. Clusters #3 and #5 scored the highest with three points accrued each; thus representing subjects with more knowledge and positive perception of cancer. Cluster #3 scored 2 points in knowledge of risk/screening and 1 in treatment/perception, while cluster #5 scored 1 in knowledge of risk/screening, 1 in treatment perception, and 1 in cancer perception. Cluster #2 had the lowest total score, scoring zero in each question category. Therefore Cluster #2 represents subjects with the least knowledge and negative perception of cancer.

**Table 4:** Knowledge and perception cluster scores

	Early detection knowledge (max score = 1)	Risk/screening knowledge (max score = 3)	Treatment perception (max score = 1)	Cancer perception (max score = 1)
**Cluster #**				
1	1	1	0	0
2	0	0	0	0
3	0	2	1	0
4	0	2	0	0
5	0	1	1	1

For the knowledge questions: >50% of subjects chose the correct answer, the score = 1, more knowledgeable

<50% of subjects chose the correct answer, the score = 0, less knowledgeable

For the perception questions: >50% of subjects answered in the affirmative, the score = 1, positive perception

<50% of subjects answered in the affirmative, the score = 0, negative perception

Table 5 then characterizes each cluster group. Cluster #2 has the highest proportion of African Americans and the lowest proportion of Whites, and the highest proportion of females and the lowest proportion of males. Cluster #3 contains the highest proportion of males and the lowest proportion of females. Cluster #5 has the highest proportion of African Americans and the lowest proportion of Whites, as well as the highest percentage of subjects that have a post-secondary education and the lowest percentage of people with a high school level education.

**Table 5:** Cluster description

	1	2	3	4	5
**Race**					
Caucasian	27.0%	**36.0%**	15.0%	17.0%	12.0%
African American	68.0%	**61.0%**	79.0%	75.0%	81.0%
Other	5.0%	3.0%	6.0%	8.0%	7.0%
					
**Education Level**					
High school or less	9.0%	---	13.0%	4.0%	**19.0%**
Post-secondary education	91.0%	100.0%	87.0%	96.0%	**81.0%**
					
**Annual Household Income**					
Less than $35,000	48.0%	57.0%	62.0%	**71.0%**	56.0%
$35,000 to $55,000	16.0%	14.0%	13.0%	**16.0%**	19.0%
More than $55,000	36.0%	29.0%	25.0%	**13.0%**	25.0%
					
**Gender**					
Male	38%	30%	41%	32%	37%
Female	62%	70%	59%	68%	63%
					
**Age**					
18–35	58%	45%	49%	**39%**	51%
36–55	23%	30%	29%	**41%**	22%
56+	19%	25%	22%	**20%**	27%

## Discussion

The results of our study show that African Americans are less knowledgeable about risk factors related to cancer occurrence, knew less about methods of treatment, and have less knowledge and more negative perceptions of clinical trials in comparison to Whites. A similar study was published in 1981 in *CA Cancer J Clin*, where a nationwide sample of 750 black American men and women were interviewed and compared with the general population in another ACS-sponsored study [7]. Results from this study found that lower income black Americans generally knew less about cancer and were less exposed to cancer information than Whites. Black Americans were found to have less knowledge of cancer's warning signals, knew little about prostate cancer, were pessimistic about methods of treating cancer, and underestimated the prevalence of cancer. Comparing our results to those of this study, some positive changes are noticed. African American men exhibit knowledge of the importance of prostate cancer by having been or expressing a want to be screened for prostate cancer in the future. African Americans also show greater knowledge of cancer risk by recognizing that minorities are at greater risk of cancer. Although not at the same magnitude, our results reflect those of the previous study 27 years later. These results highlight the great need for cancer education efforts in African American communities, particularly in underserved areas.

The lack of knowledge and poor perception of cancer is partly responsible for behaviors that can increase cancer risk in minorities. Behavioral factors have long been recognized as significant influences of cancer incidence and cancer-related outcomes [8]. There is strong evidence that reductions in cancer mortality are linked with change in behavioral risk factors, such as smoking, diet, and adherence to cancer screening tests. Some researchers estimate that if people were to follow currently available recommendations for cancer prevention and early detection, US national cancer mortality could be reduced by as much as 60% [6]. Information on reducing cancer risk is needed for African Americans that is suited to their needs and can be applied to their lifestyles. Individuals benefit from health information that reflects their cultural backgrounds, values, and belief systems [9]. For example, a study performed by Williams et al. concluded that photopictorials, or pictorial representations with captions, depicting the process of mammography for Black women were culturally acceptable and diminished women's fears and embarrassment about cancer screening [2,10]. Importantly noted in the previous study as well as in additional literature, Black Americans are interested in cancer education [7].

Teaching underserved African Americans about clinical trials may contribute to removing false beliefs about cancer and its treatment. Mistrust of clinical research and the medical profession as a whole have presented as barriers to minority, particularly African American, participation in clinical trials [11]. Educational sessions on the modalities used to treat cancer and the role of clinical trials in the current treatment of cancer would be most useful. Previous studies have demonstrated that community education and recruitment is more effective compared with that from the health care system [11]. The involvement of leaders and organizations well-respected in minority communities may help ease mistrust and may result in more cooperation and more desire to learn about and participate in clinical trials.

The cluster analysis performed on this data did not identify a specific profile that could describe knowledge and perception, other than education. A possible reason for this is the small study population. Men and women were asked if they had been or plan to be screened for prostate and breast cancer respectively. The overall response rate to these questions was low; therefore, we were unable to calculate proportions according to race while adjusted for income and education.

For a more accurate description of the attitudes of the general population, subject numbers should increase and include a more balanced representation from each demographic. For future study, additional questions pertaining to risk factors, e.g. possible engagement in behaviors that increase cancer risk and willingness to alter those behaviors, should also be explored. Perceptions of cancer information, e.g. is information relatable to subjects' lifestyle and background, to determine more effective methods of communication should be considered as well.

## Conclusion

While knowledge and positive perceptions of cancer have overall increased in the general population, underserved African Americans are still behind. Cancer prevention efforts tailored to the needs of individuals in underserved communities is a priority for public health educators.

## Competing interests

The authors declare that they have no competing interests.

## Authors' contributions

NT, CG, BK, and DEH conceived the study and, NT, CG, BK, DEH, CR, and ET participated in the study design. NT, CG, and BK developed the survey instrument, and NT, CR, and ET performed the data entry and analysis. CAS and CB were responsible for overseeing the data collection at Hampton, RJA, JAB, JCC, ATM, and DJW participated in the participant recruitment and data collection at Hampton. NT and CG participated in participant recruitment and data collection in Pittsburgh, PA. All authors read and approved the final manuscript.
